# 2-(3-Benzoyl-4-hy­droxy-1,1-dioxo-2*H*-1λ^6^,2-benzothia­zin-2-yl)-1-phenyl­ethanone

**DOI:** 10.1107/S1600536811052615

**Published:** 2011-12-10

**Authors:** Nazia Sattar, Hamid Latif Siddiqui, Syed Iftikhar Hussain Bukhari, Matloob Ahmad, Masood Parvez

**Affiliations:** a Institute of Chemistry, University of the Punjab, Lahore 54590, Pakistan; b Department of Chemistry, Government College University, Faisalabad 3800, Pakistan; c Department of Chemistry, The University of Calgary, 2500 University Drive NW, Calgary, Alberta, Canada T2N 1N4

## Abstract

In the title mol­ecule, C_23_H_17_NO_5_S, the heterocyclic thia­zine ring adopts a half-chair conformation, with the S and N atoms displaced by 0.383 (3) and 0.473 (3) Å, respectively, on opposite sides of the mean plane formed by the ring C atoms. The phenyl rings attached to carbonyl groups lie almost parallel to each other at a dihedral angle 7.43 (9)°, the distance between the centroids of the rings being 3.780 (1) Å. The C(thia­zine)—C=O and O=C—CH_2_ groups make dihedral angles of 37.56 (16) and 1.93 (18)°, respectively, with the phenyl groups to which they are attached. The crystal structure features O—H⋯O and C—H⋯O hydrogen bonds and further consolidated by C—H⋯π inter­actions; an intra­molecular O—H⋯O hydrogen bond is also present.

## Related literature

For the biological activity of benzothia­zine derivatives, see: Ahmad *et al.* (2010 ▶); Siddiqui *et al.* (2007 ▶). For related structures, see: Siddiqui *et al.* (2008 ▶).
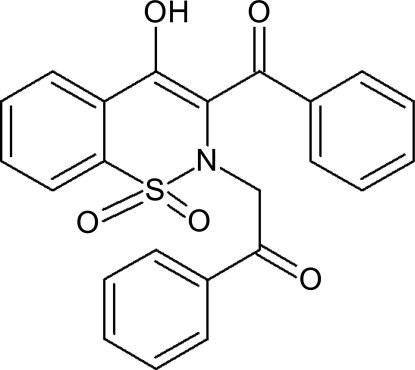


## Experimental

### 

#### Crystal data

C_23_H_17_NO_5_S
*M* *_r_* = 419.44
Triclinic, 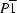

*a* = 7.5458 (2) Å
*b* = 10.9169 (4) Å
*c* = 12.0924 (4) Å
α = 101.920 (2)°
β = 101.423 (2)°
γ = 90.484 (2)°
*V* = 954.08 (5) Å^3^
*Z* = 2
Mo *K*α radiation
μ = 0.21 mm^−1^
*T* = 173 K
0.24 × 0.14 × 0.12 mm

#### Data collection

Nonius KappaCCD diffractometer
Absorption correction: multi-scan (*SORTAV*; Blessing, 1997 ▶) *T*_min_ = 0.952, *T*_max_ = 0.976
8312 measured reflections
4362 independent reflections
3706 reflections with *I* > 2σ(*I*)
*R*_int_ = 0.026

#### Refinement

*R*[*F* ^2^ > 2σ(*F* ^2^)] = 0.044
*wR*(*F* ^2^) = 0.107
*S* = 1.07
4362 reflections
272 parameters
H-atom parameters constrained
Δρ_max_ = 0.27 e Å^−3^
Δρ_min_ = −0.37 e Å^−3^

### 

Data collection: *COLLECT* (Hooft, 1998 ▶); cell refinement: *DENZO* (Otwinowski & Minor, 1997 ▶); data reduction: *SCALEPACK* (Otwinowski & Minor, 1997 ▶); program(s) used to solve structure: *SHELXS97* (Sheldrick, 2008 ▶); program(s) used to refine structure: *SHELXL97* (Sheldrick, 2008 ▶); molecular graphics: *ORTEP-3 for Windows* (Farrugia, 1997 ▶); software used to prepare material for publication: *SHELXL97*.

## Supplementary Material

Crystal structure: contains datablock(s) global, I. DOI: 10.1107/S1600536811052615/wn2461sup1.cif

Structure factors: contains datablock(s) I. DOI: 10.1107/S1600536811052615/wn2461Isup2.hkl

Supplementary material file. DOI: 10.1107/S1600536811052615/wn2461Isup3.cml

Additional supplementary materials:  crystallographic information; 3D view; checkCIF report

## Figures and Tables

**Table 1:** Hydrogen-bond geometry (Å, °) *Cg*1 is the centroid of the C10–C15 ring.

*D*—H⋯*A*	*D*—H	H⋯*A*	*D*⋯*A*	*D*—H⋯*A*
O3—H3*O*⋯O4^i^	0.84	2.43	3.026 (2)	129
C5—H5⋯O5^ii^	0.95	2.52	3.311 (2)	140
C22—H22⋯O3^iii^	0.95	2.57	3.346 (2)	139
O3—H3*O*⋯O4	0.84	1.80	2.537 (2)	146
C16—H16*B*⋯*Cg*1^iv^	0.99	2.78	3.455 (2)	126

Symmetry codes: (i) 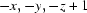; (ii) 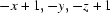; (iii) 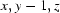; (iv) 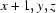.

**Fig. 1.**
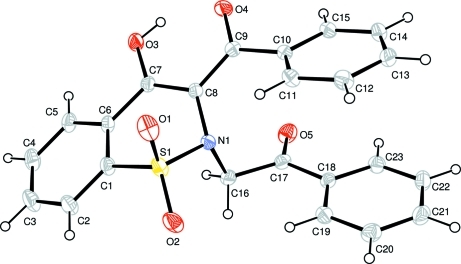
The molecular structure, with displacement ellipsoids plotted at the 30% probability level. Hydrogen atoms are shown as spheres of arbitrary radius.

**Fig. 2.**
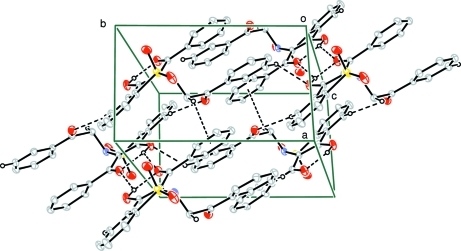
A unit cell packing diagram of the title compound showing hydrogen bonds and C—H···π-interactions drawn as dashed lines. Hydrogen atoms not involved in H-bonds have been excluded for clarity.

## supplementary crystallographic information

### Comment

In continuation of our research on the synthesis of biologically active benzothiazine derivatives (Siddiqui *et al.*, 2007 and Ahmad *et al.*, 2010), we now report the synthesis and crystal structure of the title compound.

The bond distances and angles in the title compound (Fig. 1) agree very well with the corresponding bond distances and angles reported in closely related compounds (Siddiqui *et al.*, 2008). The heterocyclic thiazine ring adopts a half-chair conformation with atoms S1 and N1 displaced by 0.383 (3) and 0.473 (3) Å, respectively, on opposite sides of the mean plane formed by the ring C atoms. The phenyl rings C10–C15 and C18–C23 lie almost parallel to each other, at a dihedral angle of 7.43 (9)°, the distance between the centroids of the rings being 3.780 (1) Å. The O4/C9/C8 and O5/C17/C16 groups are oriented at 37.56 (16) and 1.93 (18)°, respectively, with the phenyl rings to which they are bonded.

The crystal structure is stabilized by intermolecular O—H···O and C—H···O hydrogen bonds and further consolidated by C—H···π-interactions (Fig. 2); an intramolecular O—H···O hydrogen bond is also present (Table 1).

### Experimental

A mixture of 3-benzoyl-4-hydroxy-2*H*-1,2-benzothiazine 1,1-dioxide (2.5 g, 8.30 mmol) in acetone (25 ml), aqueous sodium hydroxide (0.67 g, 16.6 mmol) and 2-bromo-1-phenylethanone (1.98 g, 9.96 mmol) was subjected to ultrasonic irradiation for 20 minutes at 318 K followed by addition of HCl (5%) to maintain a pH value of 3.0. Chrome yellow precipitates of the title compound were formed, which were collected and washed with excess distilled water. Crystals suitable for crystallographic study were grown from methanol at room temperature. Yield = 3.1 g, 89.08%; m.p. = 451 - 453 K.

### Refinement

Though all the H atoms could be located in the difference Fourier map the they were included at geometrically idealized positions and refined in the riding-model approximation with the following constraints: O—H = 0.84, C—H = 0.95 and 0.99 Å for Csp^2^—H and C(methylene)—H, respectively; *U*_iso_(H) = 1.2*U*_eq_(C,O). The final difference map was essentially featurless.

### Crystal data

**Table:**

C_23_H_17_NO_5_S	*Z* = 2
*M**_r_* = 419.44	*F*(000) = 436
Triclinic, *P*1	*D*_x_ = 1.460 Mg m^−^^3^
Hall symbol: -P 1	Mo *K*α radiation, λ = 0.71073 Å
*a* = 7.5458 (2) Å	Cell parameters from 4252 reflections
*b* = 10.9169 (4) Å	θ = 1.0–27.5°
*c* = 12.0924 (4) Å	µ = 0.21 mm^−^^1^
α = 101.920 (2)°	*T* = 173 K
β = 101.423 (2)°	Block, yellow
γ = 90.484 (2)°	0.24 × 0.14 × 0.12 mm
*V* = 954.08 (5) Å^3^	

### Data collection

**Table:**

Nonius KappaCCD diffractometer	4362 independent reflections
Radiation source: fine-focus sealed tube	3706 reflections with *I* > 2σ(*I*)
graphite	*R*_int_ = 0.026
ω and φ scans	θ_max_ = 27.5°, θ_min_ = 3.0°
Absorption correction: multi-scan (*SORTAV*; Blessing, 1997)	*h* = −9→9
*T*_min_ = 0.952, *T*_max_ = 0.976	*k* = −14→14
8312 measured reflections	*l* = −15→15

### Refinement

**Table:**

Refinement on *F*^2^	Primary atom site location: structure-invariant direct methods
Least-squares matrix: full	Secondary atom site location: difference Fourier map
*R*[*F*^2^ > 2σ(*F*^2^)] = 0.044	Hydrogen site location: inferred from neighbouring sites
*wR*(*F*^2^) = 0.107	H-atom parameters constrained
*S* = 1.07	*w* = 1/[σ^2^(*F*_o_^2^) + (0.0313*P*)^2^ + 0.6186*P*] where *P* = (*F*_o_^2^ + 2*F*_c_^2^)/3
4362 reflections	(Δ/σ)_max_ < 0.001
272 parameters	Δρ_max_ = 0.27 e Å^−^^3^
0 restraints	Δρ_min_ = −0.37 e Å^−^^3^

### Special details

**Table:**

Geometry. All e.s.d.'s (except the e.s.d. in the dihedral angle between two l.s. planes) are estimated using the full covariance matrix. The cell e.s.d.'s are taken into account individually in the estimation of e.s.d.'s in distances, angles and torsion angles; correlations between e.s.d.'s in cell parameters are only used when they are defined by crystal symmetry. An approximate (isotropic) treatment of cell e.s.d.'s is used for estimating e.s.d.'s involving l.s. planes.
Refinement. Refinement of *F*^2^ against ALL reflections. The weighted *R*-factor *wR* and goodness of fit *S* are based on *F*^2^, conventional *R*-factors *R* are based on *F*, with *F* set to zero for negative *F*^2^. The threshold expression of *F*^2^ > σ(*F*^2^) is used only for calculating *R*-factors(gt) *etc*. and is not relevant to the choice of reflections for refinement. *R*-factors based on *F*^2^ are statistically about twice as large as those based on *F*, and *R*- factors based on ALL data will be even larger.

### Fractional atomic coordinates and isotropic or equivalent isotropic displacement parameters (Å^2^)

**Table:**

	*x*	*y*	*z*	*U*_iso_*/*U*_eq_	
S1	0.34149 (7)	−0.18920 (4)	0.12065 (4)	0.03505 (13)	
O1	0.1720 (2)	−0.14634 (15)	0.07084 (12)	0.0479 (4)	
O2	0.4317 (2)	−0.27840 (14)	0.05051 (12)	0.0515 (4)	
O3	0.2650 (2)	0.04686 (11)	0.43060 (11)	0.0393 (3)	
H3O	0.1841	0.0150	0.4561	0.047*	
O4	0.03228 (19)	−0.11927 (12)	0.43691 (12)	0.0417 (3)	
O5	0.3911 (2)	−0.37516 (13)	0.44672 (11)	0.0434 (3)	
N1	0.31001 (19)	−0.24683 (13)	0.23118 (12)	0.0282 (3)	
C1	0.4892 (2)	−0.05913 (16)	0.19162 (15)	0.0321 (4)	
C2	0.6247 (3)	−0.02094 (19)	0.14220 (17)	0.0416 (5)	
H2	0.6424	−0.0660	0.0695	0.050*	
C3	0.7341 (3)	0.0844 (2)	0.20105 (19)	0.0490 (5)	
H3	0.8273	0.1123	0.1682	0.059*	
C4	0.7082 (3)	0.14888 (19)	0.30697 (18)	0.0442 (5)	
H4	0.7848	0.2205	0.3467	0.053*	
C5	0.5726 (3)	0.11088 (16)	0.35621 (16)	0.0360 (4)	
H5	0.5553	0.1568	0.4287	0.043*	
C6	0.4613 (2)	0.00504 (15)	0.29926 (14)	0.0289 (4)	
C7	0.3151 (2)	−0.03730 (15)	0.35027 (14)	0.0284 (4)	
C8	0.2340 (2)	−0.15732 (15)	0.31233 (14)	0.0270 (3)	
C9	0.0816 (2)	−0.19319 (16)	0.35527 (15)	0.0299 (4)	
C10	−0.0199 (2)	−0.31649 (16)	0.30855 (15)	0.0285 (3)	
C11	−0.0556 (2)	−0.37228 (18)	0.19143 (16)	0.0335 (4)	
H11	−0.0137	−0.3318	0.1383	0.040*	
C12	−0.1522 (2)	−0.48683 (18)	0.15228 (17)	0.0383 (4)	
H12	−0.1780	−0.5240	0.0722	0.046*	
C13	−0.2109 (2)	−0.54715 (18)	0.22929 (18)	0.0377 (4)	
H13	−0.2742	−0.6267	0.2024	0.045*	
C14	−0.1775 (3)	−0.49171 (18)	0.34562 (17)	0.0374 (4)	
H14	−0.2192	−0.5328	0.3983	0.045*	
C15	−0.0836 (2)	−0.37660 (17)	0.38541 (16)	0.0326 (4)	
H15	−0.0623	−0.3384	0.4652	0.039*	
C16	0.4658 (2)	−0.31090 (16)	0.28640 (17)	0.0344 (4)	
H16A	0.5508	−0.2488	0.3443	0.041*	
H16B	0.5316	−0.3537	0.2274	0.041*	
C17	0.3936 (2)	−0.40619 (16)	0.34464 (15)	0.0311 (4)	
C18	0.3239 (2)	−0.53246 (16)	0.27569 (15)	0.0284 (4)	
C19	0.3222 (2)	−0.56942 (17)	0.15799 (16)	0.0345 (4)	
H19	0.3679	−0.5131	0.1188	0.041*	
C20	0.2540 (3)	−0.68798 (19)	0.09805 (17)	0.0397 (4)	
H20	0.2531	−0.7128	0.0179	0.048*	
C21	0.1876 (3)	−0.76998 (18)	0.15442 (18)	0.0414 (5)	
H21	0.1403	−0.8510	0.1129	0.050*	
C22	0.1894 (3)	−0.73471 (18)	0.27146 (18)	0.0398 (4)	
H22	0.1439	−0.7915	0.3103	0.048*	
C23	0.2577 (2)	−0.61663 (17)	0.33150 (16)	0.0345 (4)	
H23	0.2594	−0.5927	0.4118	0.041*	

### Atomic displacement parameters (Å^2^)

**Table:**

	*U*^11^	*U*^22^	*U*^33^	*U*^12^	*U*^13^	*U*^23^
S1	0.0427 (3)	0.0347 (2)	0.0262 (2)	−0.01221 (19)	0.01089 (18)	−0.00028 (17)
O1	0.0480 (8)	0.0589 (9)	0.0339 (7)	−0.0119 (7)	−0.0025 (6)	0.0141 (7)
O2	0.0661 (10)	0.0448 (8)	0.0429 (8)	−0.0181 (7)	0.0321 (7)	−0.0112 (6)
O3	0.0554 (9)	0.0261 (6)	0.0396 (7)	−0.0039 (6)	0.0237 (6)	0.0008 (5)
O4	0.0470 (8)	0.0337 (7)	0.0467 (8)	−0.0002 (6)	0.0265 (6)	−0.0024 (6)
O5	0.0555 (9)	0.0369 (7)	0.0334 (7)	0.0010 (6)	0.0065 (6)	0.0000 (6)
N1	0.0310 (7)	0.0238 (7)	0.0303 (7)	−0.0026 (6)	0.0123 (6)	0.0015 (6)
C1	0.0388 (9)	0.0280 (9)	0.0287 (8)	−0.0073 (7)	0.0077 (7)	0.0037 (7)
C2	0.0474 (11)	0.0401 (11)	0.0390 (10)	−0.0107 (9)	0.0180 (9)	0.0043 (8)
C3	0.0497 (12)	0.0487 (12)	0.0508 (12)	−0.0194 (10)	0.0169 (10)	0.0100 (10)
C4	0.0491 (12)	0.0352 (10)	0.0451 (11)	−0.0170 (9)	0.0047 (9)	0.0064 (9)
C5	0.0478 (11)	0.0262 (9)	0.0319 (9)	−0.0061 (8)	0.0052 (8)	0.0045 (7)
C6	0.0361 (9)	0.0236 (8)	0.0275 (8)	−0.0019 (7)	0.0060 (7)	0.0075 (6)
C7	0.0364 (9)	0.0241 (8)	0.0264 (8)	0.0018 (7)	0.0094 (7)	0.0060 (6)
C8	0.0310 (8)	0.0243 (8)	0.0265 (8)	0.0004 (7)	0.0092 (7)	0.0043 (6)
C9	0.0316 (9)	0.0273 (8)	0.0312 (9)	0.0030 (7)	0.0083 (7)	0.0056 (7)
C10	0.0245 (8)	0.0272 (8)	0.0344 (9)	0.0012 (6)	0.0086 (7)	0.0056 (7)
C11	0.0295 (9)	0.0384 (10)	0.0326 (9)	−0.0041 (7)	0.0067 (7)	0.0072 (7)
C12	0.0328 (9)	0.0411 (11)	0.0361 (10)	−0.0051 (8)	0.0035 (8)	0.0007 (8)
C13	0.0310 (9)	0.0298 (9)	0.0513 (11)	−0.0022 (7)	0.0082 (8)	0.0066 (8)
C14	0.0369 (10)	0.0345 (10)	0.0460 (11)	−0.0003 (8)	0.0142 (8)	0.0151 (8)
C15	0.0316 (9)	0.0343 (9)	0.0338 (9)	0.0009 (7)	0.0115 (7)	0.0070 (7)
C16	0.0293 (9)	0.0271 (9)	0.0462 (10)	0.0002 (7)	0.0117 (8)	0.0023 (8)
C17	0.0278 (8)	0.0286 (9)	0.0347 (9)	0.0049 (7)	0.0042 (7)	0.0042 (7)
C18	0.0264 (8)	0.0261 (8)	0.0326 (9)	0.0031 (6)	0.0058 (7)	0.0058 (7)
C19	0.0364 (9)	0.0333 (9)	0.0348 (9)	−0.0006 (7)	0.0105 (8)	0.0066 (7)
C20	0.0420 (11)	0.0390 (10)	0.0340 (10)	0.0011 (8)	0.0074 (8)	−0.0010 (8)
C21	0.0365 (10)	0.0301 (9)	0.0519 (12)	−0.0016 (8)	0.0047 (9)	0.0006 (8)
C22	0.0389 (10)	0.0318 (10)	0.0507 (12)	−0.0019 (8)	0.0084 (9)	0.0137 (9)
C23	0.0361 (9)	0.0359 (10)	0.0332 (9)	0.0030 (8)	0.0075 (8)	0.0109 (8)

### Geometric parameters (Å, °)

**Table:**

S1—O2	1.4249 (15)	C10—C15	1.396 (2)
S1—O1	1.4281 (16)	C11—C12	1.386 (2)
S1—N1	1.6460 (15)	C11—H11	0.9500
S1—C1	1.7566 (17)	C12—C13	1.382 (3)
O3—C7	1.309 (2)	C12—H12	0.9500
O3—H3O	0.8400	C13—C14	1.383 (3)
O4—C9	1.259 (2)	C13—H13	0.9500
O5—C17	1.215 (2)	C14—C15	1.383 (3)
N1—C8	1.443 (2)	C14—H14	0.9500
N1—C16	1.488 (2)	C15—H15	0.9500
C1—C2	1.384 (2)	C16—C17	1.523 (3)
C1—C6	1.401 (2)	C16—H16A	0.9900
C2—C3	1.387 (3)	C16—H16B	0.9900
C2—H2	0.9500	C17—C18	1.487 (2)
C3—C4	1.379 (3)	C18—C23	1.390 (2)
C3—H3	0.9500	C18—C19	1.394 (2)
C4—C5	1.382 (3)	C19—C20	1.385 (3)
C4—H4	0.9500	C19—H19	0.9500
C5—C6	1.393 (2)	C20—C21	1.378 (3)
C5—H5	0.9500	C20—H20	0.9500
C6—C7	1.479 (2)	C21—C22	1.385 (3)
C7—C8	1.389 (2)	C21—H21	0.9500
C8—C9	1.434 (2)	C22—C23	1.381 (3)
C9—C10	1.488 (2)	C22—H22	0.9500
C10—C11	1.392 (2)	C23—H23	0.9500
			
O2—S1—O1	119.49 (10)	C12—C11—H11	120.0
O2—S1—N1	108.46 (9)	C10—C11—H11	120.0
O1—S1—N1	107.29 (8)	C13—C12—C11	120.28 (18)
O2—S1—C1	110.05 (9)	C13—C12—H12	119.9
O1—S1—C1	109.15 (9)	C11—C12—H12	119.9
N1—S1—C1	100.71 (8)	C12—C13—C14	119.98 (17)
C7—O3—H3O	109.5	C12—C13—H13	120.0
C8—N1—C16	113.65 (14)	C14—C13—H13	120.0
C8—N1—S1	111.94 (11)	C13—C14—C15	120.22 (17)
C16—N1—S1	115.61 (11)	C13—C14—H14	119.9
C2—C1—C6	121.76 (16)	C15—C14—H14	119.9
C2—C1—S1	121.28 (14)	C14—C15—C10	120.14 (17)
C6—C1—S1	116.95 (13)	C14—C15—H15	119.9
C1—C2—C3	118.64 (18)	C10—C15—H15	119.9
C1—C2—H2	120.7	N1—C16—C17	108.46 (14)
C3—C2—H2	120.7	N1—C16—H16A	110.0
C4—C3—C2	120.36 (18)	C17—C16—H16A	110.0
C4—C3—H3	119.8	N1—C16—H16B	110.0
C2—C3—H3	119.8	C17—C16—H16B	110.0
C3—C4—C5	120.99 (17)	H16A—C16—H16B	108.4
C3—C4—H4	119.5	O5—C17—C18	121.73 (17)
C5—C4—H4	119.5	O5—C17—C16	118.41 (16)
C4—C5—C6	119.86 (17)	C18—C17—C16	119.84 (15)
C4—C5—H5	120.1	C23—C18—C19	118.96 (16)
C6—C5—H5	120.1	C23—C18—C17	118.26 (16)
C5—C6—C1	118.39 (16)	C19—C18—C17	122.78 (16)
C5—C6—C7	120.79 (16)	C20—C19—C18	120.18 (17)
C1—C6—C7	120.81 (15)	C20—C19—H19	119.9
O3—C7—C8	122.77 (15)	C18—C19—H19	119.9
O3—C7—C6	115.46 (14)	C21—C20—C19	120.17 (18)
C8—C7—C6	121.75 (15)	C21—C20—H20	119.9
C7—C8—C9	121.00 (15)	C19—C20—H20	119.9
C7—C8—N1	118.45 (14)	C20—C21—C22	120.25 (18)
C9—C8—N1	120.52 (14)	C20—C21—H21	119.9
O4—C9—C8	119.42 (15)	C22—C21—H21	119.9
O4—C9—C10	118.06 (15)	C23—C22—C21	119.70 (18)
C8—C9—C10	122.51 (15)	C23—C22—H22	120.2
C11—C10—C15	119.30 (16)	C21—C22—H22	120.2
C11—C10—C9	122.38 (16)	C22—C23—C18	120.75 (17)
C15—C10—C9	118.31 (15)	C22—C23—H23	119.6
C12—C11—C10	120.05 (17)	C18—C23—H23	119.6
			
O2—S1—N1—C8	174.71 (11)	S1—N1—C8—C9	132.02 (14)
O1—S1—N1—C8	−54.93 (13)	C7—C8—C9—O4	−7.3 (3)
C1—S1—N1—C8	59.18 (13)	N1—C8—C9—O4	170.73 (16)
O2—S1—N1—C16	42.43 (14)	C7—C8—C9—C10	173.95 (16)
O1—S1—N1—C16	172.79 (12)	N1—C8—C9—C10	−8.0 (3)
C1—S1—N1—C16	−73.10 (13)	O4—C9—C10—C11	142.68 (18)
O2—S1—C1—C2	30.8 (2)	C8—C9—C10—C11	−38.5 (3)
O1—S1—C1—C2	−102.15 (18)	O4—C9—C10—C15	−36.2 (2)
N1—S1—C1—C2	145.15 (17)	C8—C9—C10—C15	142.54 (18)
O2—S1—C1—C6	−150.24 (15)	C15—C10—C11—C12	−0.5 (3)
O1—S1—C1—C6	76.79 (16)	C9—C10—C11—C12	−179.43 (17)
N1—S1—C1—C6	−35.90 (16)	C10—C11—C12—C13	−1.0 (3)
C6—C1—C2—C3	−0.6 (3)	C11—C12—C13—C14	1.7 (3)
S1—C1—C2—C3	178.33 (17)	C12—C13—C14—C15	−0.7 (3)
C1—C2—C3—C4	0.4 (3)	C13—C14—C15—C10	−0.8 (3)
C2—C3—C4—C5	−0.5 (4)	C11—C10—C15—C14	1.5 (3)
C3—C4—C5—C6	0.8 (3)	C9—C10—C15—C14	−179.59 (16)
C4—C5—C6—C1	−1.0 (3)	C8—N1—C16—C17	74.17 (17)
C4—C5—C6—C7	−179.94 (18)	S1—N1—C16—C17	−154.35 (12)
C2—C1—C6—C5	0.9 (3)	N1—C16—C17—O5	−95.19 (19)
S1—C1—C6—C5	−178.09 (14)	N1—C16—C17—C18	83.21 (18)
C2—C1—C6—C7	179.81 (18)	O5—C17—C18—C23	−1.1 (3)
S1—C1—C6—C7	0.9 (2)	C16—C17—C18—C23	−179.48 (16)
C5—C6—C7—O3	18.8 (2)	O5—C17—C18—C19	178.78 (18)
C1—C6—C7—O3	−160.17 (17)	C16—C17—C18—C19	0.4 (3)
C5—C6—C7—C8	−163.06 (17)	C23—C18—C19—C20	0.5 (3)
C1—C6—C7—C8	18.0 (3)	C17—C18—C19—C20	−179.41 (17)
O3—C7—C8—C9	3.8 (3)	C18—C19—C20—C21	0.0 (3)
C6—C7—C8—C9	−174.28 (16)	C19—C20—C21—C22	−0.4 (3)
O3—C7—C8—N1	−174.30 (16)	C20—C21—C22—C23	0.2 (3)
C6—C7—C8—N1	7.7 (2)	C21—C22—C23—C18	0.3 (3)
C16—N1—C8—C7	83.33 (19)	C19—C18—C23—C22	−0.7 (3)
S1—N1—C8—C7	−49.91 (19)	C17—C18—C23—C22	179.26 (17)
C16—N1—C8—C9	−94.73 (18)		

### Hydrogen-bond geometry (Å, °)

**Table:**

Cg1 is the centroid of the C10–C15 ring.

**Table:**

*D*—H···*A*	*D*—H	H···*A*	*D*···*A*	*D*—H···*A*
O3—H3O···O4^i^	0.84	2.43	3.026 (2)	129
C5—H5···O5^ii^	0.95	2.52	3.311 (2)	140
C22—H22···O3^iii^	0.95	2.57	3.346 (2)	139
O3—H3O···O4	0.84	1.80	2.537 (2)	146
C16—H16B···Cg1^iv^	0.99	2.78	3.455 (2)	126

Symmetry codes: (i) −*x*, −*y*, −*z*+1; (ii) −*x*+1, −*y*, −*z*+1; (iii) *x*, *y*−1, *z*; (iv) *x*+1, *y*, *z*.
